# Specialized Medical Weight Management Intervention for High-Risk Obesity

**DOI:** 10.36469/001c.24896

**Published:** 2021-07-01

**Authors:** Gitanjali Srivastava, Chelsea Paris, Jessica Johnson, Emma Barnes, Brittany L. Cunningham, C. J. Stimson, Kevin D. Niswender, Sabrina J. Poon

**Affiliations:** 1 Department of Medicine, Division of Diabetes, Endocrinology & Metabolism, Vanderbilt University School of Medicine, Nashville, TN; Department of Surgery and Department of Finance, Vanderbilt University Medical Center, Nashville, TN; 2 Department of Finance Vanderbilt University Medical Center, Nashville, TN; 3 Office of Episodes of Care, Office of Population Health Vanderbilt University Medical Center, Nashville, TN; 4 Office of Episodes of Care, Office of Population Health and Department of Urology Vanderbilt University Medical Center, Nashville, TN; 5 Department of Medicine, Division of Diabetes, Endocrinology & Metabolism Vanderbilt University School of Medicine, Nashville, TN; 6 Office of Episodes of Care, Office of Population Health and Department of Emergency Medicine Vanderbilt University Medical Center, Nashville, TN

**Keywords:** finance, weight loss outcomes, fee for service, bundle, weight management services, obesity care path

## Abstract

**Background:** Bundled payments are services rendered at pre-determined costs with the goal of providing high value care. Our institution’s Episodes of Care team partnered with its tertiary care obesity center to design a novel medical weight management bundle for employers that would collectively deliver high value obesity services.

**Objective:** As a first step, we sought to evaluate short-term medical weight loss outcomes over 6 months at the obesity center.

**Methods:** We retrospectively analyzed weight loss outcomes on 157 patients with commercial insurance coverage over a period of 6 months.

**Results:** Patients ranged in age from 18-72 years, and 77.7% were female. Patients ranged in weight from 160-443 pounds, with a mean body mass index (BMI) of 42.7 kg/m2 (Class 3a severe obesity; BMI range 28.4-74.5). The prevalence of any obesity-related medical condition was 54.1%; at least a quarter of the patients had either prediabetes or Type 2 diabetes mellitus, approximately a third had hypertension, and over 8% had hyperlipidemia. Mean weight loss from the initial program start date was 6.28% (+/-0.48% standard error of mean [SEM]; 95% confidence interval [CI] 5.34-7.23%). Completers (defined as having at least 6 visits with a medical provider) achieved a higher percentage of weight loss (7.06%) from the initial program start compared to non-completers (4.68%; at least 4-5 visits with a medical provider; *P*<0.0158). Approximately 50% of patients were able to achieve >7% weight loss, with over 55% of patients achieving at least 3% weight loss or higher irrespective of BMI classification.

**Conclusions:** Specialized medical weight intervention is effective in treating high-risk obesity with complications. This has implications for enhanced long-term cost savings related to employer coverage of such programs for their employees with obesity.

## INTRODUCTION

Obesity is a chronic, debilitating disease worldwide, exacerbating or causing more than 200 medical disorders including cardiovascular disease, diabetes, sleep apnea, and psychosocial conditions.^1–3^ An estimated US$2 trillion are attributed to it globally.^4^ A diagnosis of obesity is associated with higher rates of disability claims due to all causes, particularly psychiatric, musculoskeletal, circulatory and malignancy.^5^ Because obesity results in a significant financial burden to hospital systems, employers and patients, effective treatment is paramount in reducing new-onset or worsening comorbidities, medication costs, and hospitalizations. Recently, substantial all-cause health-care cost savings have been observed for short-term nonsurgical weight loss, including similar observations for sustained weight loss in adults with obesity (approximately US$135 per patient per month in persons with obesity if weight loss of 5-10% occurs).^6^ This might equate into economical savings for employer groups insuring a high-risk employee population. Thus, there is a financial incentive to develop obesity care paths that appropriately and effectively treat the disease state.

Our academic institution has a tertiary care multidisciplinary medical weight loss (MWL) center for patients with obesity (BMI >30 kg/m^2^) comprised of fellowship-trained obesity medicine specialists, bariatric surgeons, dietitians, behavioral health, and exercise specialists. The MWL center utilizes a multimodal approach encompassing foundational intensive lifestyle therapies (group and individual visits with dietitians and licensed social workers and medical fitness consultation) coupled with a comprehensive obesity medicine assessment that may prompt anti-obesity medications considerations (monotherapy or in combination; phentermine, phentermine/topiramate, liraglutide, naltrexone SR/buproprion SR, orlistat; off label oral or injectable semaglutide, dulaglutide, zonisamide, metformin) to treat complicated obesity. Patients are either self-referred or provider-referred typically from primary care, endocrinology, transplant services, orthopedics, or fertility clinics, as examples. These patients have not previously responded favorably to self-implemented or commercially-based weight management programs and may not be candidates presently for metabolic and bariatric surgery for numerous reasons (patient preference, insurance non-coverage of bariatric surgery, ineligible or not meeting bariatric surgery criteria, or medical condition contraindication).

The Episodes of Care and MWL teams at Vanderbilt University Medical Center set out to design a weight management bundle model for regional and local employers wishing to provide obesity coverage for their employees. “Bundled payments” are the renumeration to health-care providers for rendered services based on expected costs and quality of care.^7–10^ They provide incentives to reduce waste and overuse, coordinate care across multiple settings while delivering evidence-based, high-quality needed care to patients. The bundle, in essence, “bundles” services collectively ensuring high quality and economic value at a lower cost to the employer. Creation of an obesity bundle has never been reported in the literature previously, and is an innovative and challenging health-care delivery model. In designing a novel weight management bundle, we first sought to determine the effectiveness of standardized routine clinical care in patients with obesity being evaluated at our institution’s MWL center over a 6-month period. Due to lack of coverage of bariatric surgery and obesity services in many of the state’s insurance plans, the MWL center is predominantly comprised of patients with commercial insurance.

## METHODS

The Episodes of Care team met with the MWL team monthly for one year to research standard clinic operation flow and structure. To determine the MWL program’s efficacy, we retrospectively reviewed weight loss outcomes and utilization data from September 2019 to March 2020 for patients with commercial insurance who had at least one follow-up visit 4 months after the initial MWL visit. In addition, we examined weight loss outcomes for patients who made monthly visits with a medical obesity provider (physician or nurse practitioners in the MWL center) for a period of 6 months (at least 6 visits; completers) and for those with less frequent visits (4-5 visits; non-completers). Initial anthropometrics were defined by the measured weight and height entered into the electronic health record at the first visit with the MWL provider. End anthropometries were defined by the 6-month weight entered into the electronic health record for completers. For non-completers, end weight was defined as the last measurement at visit 4-5 in the system as no further data was available due to attrition. De-identified data was extracted through informatics from the electronic health record and then analyzed manually, as part of a quality improvement project. Manual chart review confirmed (1) all commercially insured payers, (2) no evidence of evidence of bariatric surgery considerations within 6 months of the initial visit (potential bariatric surgery patients would qualify for a different bundle for bariatric surgery and thus would be excluded from the MWL bundle), and (3) insufficient sample size of Medicaid and/or Medicare patients (n=0).

## RESULTS

A total of 157 patients (ages 18-72 years; 77.7% female; weight range 160-443 pounds) were included in the analysis (**Table 1**). Patients had a mean BMI of 42.7 kg/m^2^ (Class 3a severe obesity; BMI range 28.4-74.5). The prevalence of any obesity-related medical condition was 54.1% (depression: 5.7%; hyperlipidemia: 8.3%; hypertension: 29.9%; prediabetes: 10.1%; type 2 diabetes mellitus: 14.6%; vitamin D deficiency: 13.3%). Mean weight loss from the initial program start date was 6.28% (+/-0.48% standard error of mean [SEM]; 95% confidence interval [CI] 5.34-7.23%). In patients who had at least 6 months of visit activity (n=106), average weight loss increased to 7.06% (+/-0.60% SEM; 95% CI 5.89-8.23%; 7.022% BMI reduction +/-0.60% SEM; 95% CI 5.84-8.19%); in those with 4-5 months of visit activity (n=51), average weight loss decreased to 4.68% (+/-0.77% SEM; 95% CI 3.17-7.85%; 4.66% BMI reduction +/-0.76% SEM; 95% CI 3.16-7.82%).

**Figure 1.**
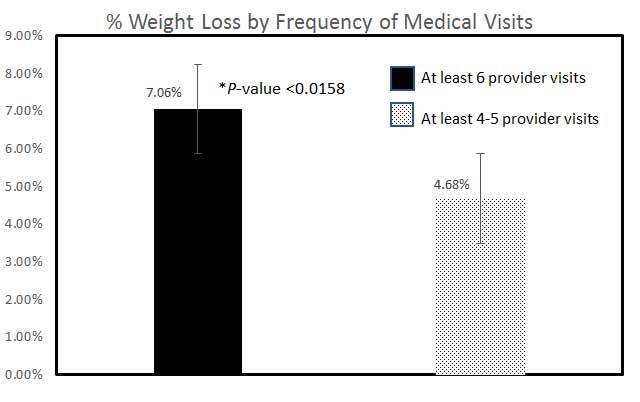
Percentage Weight Loss by Frequency of Medical Visits Figure 1 depicts % weight loss by frequency of medical provider visits (physician or nurse practitioner) at the obesity center. Patients who completed at least 6 visits (completers; n=106) had higher weight loss compared to noncompleters (at least 4-5 visits; n=51)[P<0.0158; 7.06% + 0.60 SEM vs 4.68% +0.77 SEM].

**Figure 2.**
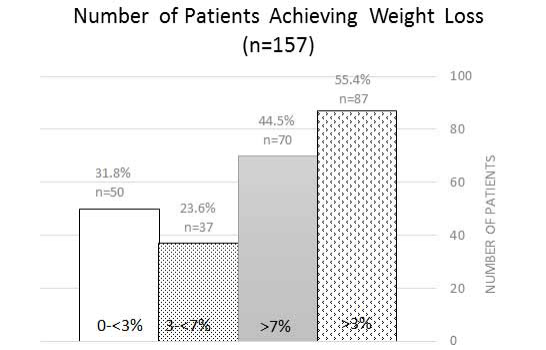
Number of Patients Achieving Weight Loss Figure 2 depicts number of patients (n=157) achieving weight loss at 6 months from baseline initial first visit, by weight loss percentage categories (patients achieving 0-< 3%, 3-<7%, >7%, >3% weight loss).

Completers achieved a higher percentage weight loss from the initial program start compared to non-completers (**Figure 1**; *P*<0.0158). This was consistent regardless of the MWL provider seen. There was no statistically significant difference between age, gender, or initial BMI among completers and non-completers (*P*>0.05). Weight loss was similar between completers and non-completers irrespective of the presence or the absence of pre-existing medical conditions (*P*=0.5863): depression, hyperlipidemia, hypertension, prediabetes, Type 2 diabetes, or vitamin D deficiency status. There was also a statistically significant difference only at the 10% level, but not at the 5% level, between the total number of anti-obesity medications prescribed between completers versus non-completers (*P*=0.0553); many of the completers were on combination anti-obesity pharmacotherapy. Approximately 50% of patients were able to achieve >7% weight loss with over 55% of patients achieving at least 3% weight loss or higher irrespective of BMI classification (**Figure 2**). The total number of anti-obesity medications prescribed between poor responders (0-< 3% total weight loss) versus supra-responders (>7% total weight loss; *P*=0.0661) was statistically significant at the 10% level, but not at the 5% level.

**Table 1.** Summary of Weight Loss Outcomes

	**Completers (at least 6 visits with medical provider)**	**Non-Completers (only 4-5 visits with medical provider)**	**Composite**
**n**	106	51	157
**Age**	44.8	43.7	43.9
**Gender (n=female)**	81	41	122
**Initial BMI Mean [BMI Range 28.4- 74.5; Weight (lbs) range 160-443]**	42.4	43.3	42.7
**Prevalence Comorbidity (any)**	55.7%	50.9%	54.1%
Depression	8.4%	1.9%	5.7%
Hyperlipidemia	7.8%	8.5%	8.3%
Hypertension	10.4%	70.6%	29.9%
Prediabetes	5.7%	19.6%	10.1%
Type 2 Diabetes Mellitus	7.5%	29.4%	14.6%
Vitamin D Deficiency	9.4%	21.6%	13.3%
Mean Weight Loss* at 6 Months or at Last Visit [SEM +/- CI]	7.06% [+/-0.60% SEM; 95% CI 5.89-8.23%]	4.68% [+/-0.77% SEM; 95% CI 3.17-7.85%]	6.28% [+/-0.48% SEM; 95% CI 5.34-7.23%]
BMI Reduction* [SEM +/- CI]	7.022% [+/-0.60% SEM; 95% CI 5.84-8.19%]	4.66% [+/-0.76% SEM; 95% CI 3.16-7.82%]	6.25% [+/-0.48 SEM; 95% CI 5.31-7.20]

* Statistically significant difference at the 5% level between completers and non-completers.

## DISCUSSION

Our MWL center’s supervised, short-term specialized weight management intervention was able to achieve a clinically meaningful weight loss outcome at 6 months (>5%) and was highly effective in treating severe obesity with related comorbidities (Class 2 or higher, >6%). These early findings are particularly striking and prompt further investigation. Small amounts of weight loss (<5%) can prevent progression to type 2 diabetes; even modest weight loss of about 5% is associated with improvement in blood pressure, and HDL lowering; more weight loss produces more improvement.^11^ The program likely leads to overall improvement in health, though more robust long-term data is needed.

The program is expected to lead to overall cost savings for the employer. Results (**Figure 2**) indicate that out of 157 patients, 87 achieved >3% weight loss and that the majority of patients had Class 2 obesity or higher based on demographics. The estimated cost per person per month savings for nonsurgical treatment of Class 2 obesity within the first year by at least 3% weight reduction is US$143.43.^6^ Based on these extrapolations, the program (n=157) would result in US$12 478.41 cost savings per month, or US$74 870.46 in 6 months. As another example, consider a large employer with an employee base of 1000. Assuming a ~40% obesity rate (Class 1 or higher),^12^ approximately 400 patients would qualify for MWL with literature reported cost savings of US$135.35 per person per month (Class 1 or higher)^6^ for nonsurgical weight loss reduction of 5-7%. This would translate to an estimated US$24 092.30 per month or US$144 553.80 in 6-month health- care cost savings for the 44.5% of patients who would be predicted to lose >7% of weight loss with treatment (**Figure 2**). It should be noted that while these are extrapolations, we plan to analyze the impact of the obesity bundle on health-care costs for the employers who enroll.

The effectiveness of the comprehensive program likely stems from the intensive support provided to each individual patient and the multidisciplinary component to address the multifactorial etiology of obesity. Treatment of obesity may be deemed difficult by both patients and providers due to numerous barriers (behavioral, environmental, psychosocial, biological and/or genetic) required to overcome in order to have a successful response. Expertise and specialized training in the management of obesity leading to improved communication, mental health and behavioral modification, adherence, and utilization of novel strategies and approaches such as pharmacotherapeutics are likely to improve patient success long-term. It should be noted that the program included fellowship trained obesity medicine specialists certified through the American Board of Obesity Medicine,^13^ underscoring the value of obesity medicine fellowship programs affiliated with institutions.^14,15^ Anti-obesity pharmacotherapy has been shown to result in net pharmaceutical cost savings compared to treating obesity related comorbid conditions such as hypertension, hyperlipidemia and diabetes.^16^ Current anti-obesity pharmacotherapy, clinically indicated in patients with a BMI >27 kg/m^2^ with an obesity-related medical condition or a BMI >30 kg/m^2^, in combination with lifestyle therapy, have an efficacy of 3-7% reduction in weight loss.^1^ Patients seeking tertiary care obesity evaluation at an academic institution are also more likely to require increased accountability, structure and resources coupled with reiteration of recommendations by the medical provider and ancillary team. Similar multidisciplinary models of obesity care have also shown comparable weight loss outcomes.^14^ Incentives should be provided to both employers and hospital organizations to invest in such obesity care paths.

While the results are encouraging, there are limitations and further research in weight loss health outcomes is much needed. We did not have consistent reportable data available on improved laboratory re-assessment following weight loss in patients. MWL provider prescription of anti-obesity medications was variable and standardized algorithms likely need to be applied in order to extrapolate pharmacy benefits into a bundle service model. We were only able to extract pharmacy data from prescriptions filled specifically at our institution and not at outside pharmacies. Of note, though there was an inclination of combination (>2 anti-obesity medications prescribed) in high responders (>7% weight loss; n=70), results were not statistically significant when comparing to poor responders (0-< 3% weight loss; n=50) at the 5% level and a larger cohort of patients is likely needed to make statistically significant correlations. Furthermore, patients with more severe obesity (BMI >35 kg/m^2^) benefited from the program; this weight loss benefit might translate to reduced overall health expenditures and cost savings in the long term for a chronic health condition. Thus the program is preferable for this high-risk population requiring the augmented structure and resources compared to patients who may need sole nutritional advice and simple guidance. Frequency of visits impacted weight loss outcomes, as patients who attended all of their monthly visits achieved greater weight loss compared to those who did not. Due to the time commitment and intensity of the visits, some patients might not be able to complete such a comprehensive program leading to inflated attrition rates. More research regarding reasons for attrition needs to be explored. Interventions to decrease attrition, such as implementing telehealth services, could improve outcomes even further and could easily be included as part of a medical weight management bundle. It should also be noted that 77% of the population cohort in the study were women. In future studies, it would be valuable to consider pregnancy-delivery (C-section vs vaginal) history, menopause status, marital status, race, and ethnicity. Though these data were not collected in this study, these may be possible confounders in the study design. Finally, this was a population of commercially insured patients, and our findings may not be generalizable. Studies have shown an association between lower socioeconomic status (which might be linked to food stamp or individual factors contributing to poor diet as examples), insurance type, and obesity.^17–20^ The impact of a nonsurgical comprehensive weight management program on this specific cohort of patients on public insurance warrants attention.

## CONCLUSIONS

As the obesity epidemic continues to rise, hospital systems are forced to think creatively and constructively to create effective obesity care paths. Multidisciplinary obesity care programs embedded within larger hospital networks provide evidence to combat severe obesity, thereby generating high cost-savings to patients and employers through weight loss. It becomes increasingly necessary to recognize the value that high quality obesity care brings to an organization and support endeavors to bring fruition to those obesity care paths.

### DISCLOSURES

GS reports advisory/consultant fees from Novo Nordisk and Rhythm, outside the submitted work. GS is a Diplomate of the American Board of Obesity Medicine. CP, JJ, EB, BLC, CJS, KDN, and SJP have nothing to disclose.

### CONTRIBUTIONS

GS conceptualized, wrote and edited the manuscript. CP participated in the design of the study, retrospective chart review and carried out the statistical analysis. All authors helped the design, coordination, drafting and editing of the manuscript.
